# A Tale of Two Kitchens, Meals and Microbes

**DOI:** 10.3201/eid2406.AC2406

**Published:** 2018-06

**Authors:** Byron Breedlove, Martin I. Meltzer

**Affiliations:** Centers for Disease Control and Prevention, Atlanta, Georgia, USA

**Keywords:** art science connection, emerging infectious diseases, art and medicine, about the cover, Vincenzo Campi, Kitchen (Cucina), a tale of two kitchens, meals, and microbes, bacteria, zoonotic diseases, zoonoses, foodborne diseases, food safety, public health

**Figure:**
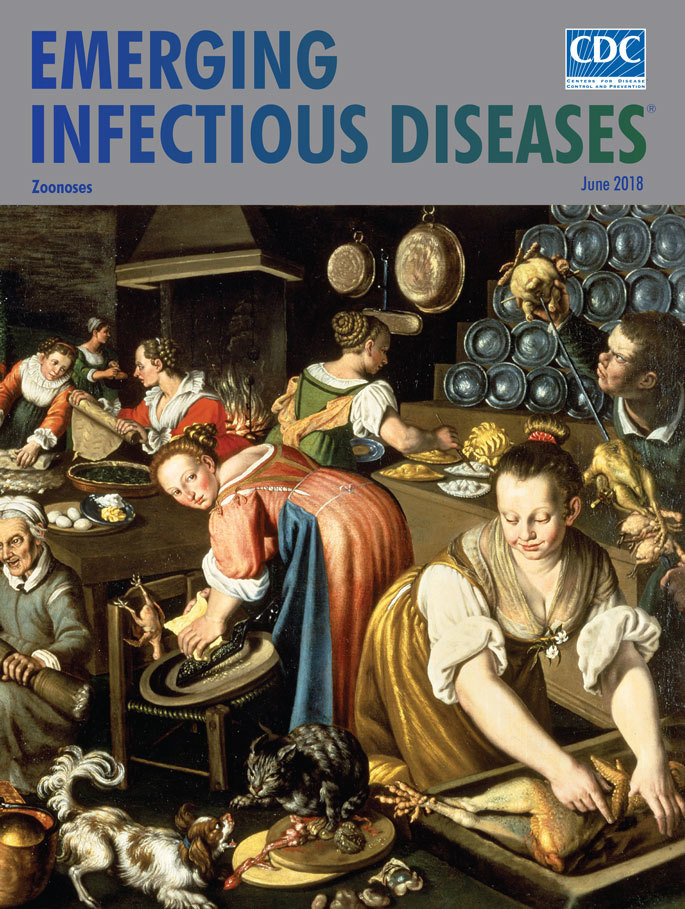
**Vincenzo Campi (1530/1535–1591), Kitchen (Cucina) (1580).** Oil on canvas, 57 in × 87 in/145 cm × 220 cm. Pinacoteca di Brera, Milan, Italy.

*Kitchen*, a painting completed in 1580 by Italian artist Vincenzo Campi, celebrates the chaotic workspace that was devoted to keeping a noble family’s house supplied with food and drink. The kitchen workers are preparing an assortment and quantity of meats, pies and breads, sauces, and side dishes as a special meal for a celebration or holiday.

Invisible to the viewer and unknown to Campi, his subjects, or his patrons, this kitchen would have been permeated by numerous unwelcome microorganisms that could cause zoonotic foodborne diseases. Such a setting would provide many opportunities for transmission of potentially pathogenic bacteria, viruses, and parasites in the raw meat and poultry, in the blood and viscera spattered on the workers’ skin and clothing, on floors and shared work surfaces, knives, and other utensils, and from domestic pets, rodents, and insects. 

Campi’s *Kitchen* is alive with activity. Near the top of the painting, demonstrating the artist’s mastery of the technique of perspective, the viewer sees a dining room containing a long table festooned with a white tablecloth and tended by a young girl. Half a dozen colorfully dressed women are busy preparing the food, seemingly using every available surface. An older woman is working on the floor and appears to react negatively to the taste or smell of whatever is covering the bottom of a large pestle. A small child is sitting on a colander, amusing himself by inflating an animal’s stomach. On the upper left periphery, a pair of men are butchering a deer carcass, while across the kitchen, a young man is carefully skewering raw, uncooked game birds on a spit. A cat and dog scrap for entrails plucked from the poultry carcass in the foreground, cooking pans dangle near rows of stacked plates in the upper right, and a small fire smolders in the fireplace near the center.

In the second half of the 16th century, Vincenzo Campi and his brothers, Giulio and Antonio, were considered among the finest artists in the northern Italy town of Cremona. Specific details about Campi’s homelife and education during the early years of his life are scarce. His father, Galeazzo Campi, also an artist of note, had been a pupil of the painter Boccaccio Boccaccini and helped educate his trio of sons in the arts. Giulio, the eldest brother, was a noted architect and artist, who also instructed his younger siblings.

A short biography from the Museo Del Prado notes that Campi’s earliest collaborations with his brothers showed little originality. His initial efforts were chiefly portraits of members of the upper class and various Catholic saints. Although throughout his career Campi continued to paint religious iconography and portraits for wealthy patrons, he is remembered more for his realistic paintings that captured the bustle of everyday life among the lower economic classes, food merchants, poultry and fish vendors, butchers, cooks, and kitchen workers.

Sheila McTighe, senior lecturer at the Courtauld Institute of Art, stated that Campi “is best known for his significant contribution to the birth of northern Italian genre painting. The style appeared quite suddenly between 1580 and 1585 in Cremona and Bologna, and its development was heavily influenced by similar genre paintings by Flemish artists Pieter Aertsen and Joachim Beuckelaer.” Wealthy merchants and bankers—some of whom were no doubt the subjects of Campi’s portraiture—imported examples of those Flemish genre paintings to northern Italy, and Campi would have had ready access to them. Exactly what drove the sudden demand for representational art is not clear.

Art scholar Deborah Krohn notes that “Kitchens, along with foodstuffs, do not appear as a significant focus in paintings until the middle of the sixteenth century. First in the Low Countries, and then in Italy, we find kitchens as the settings for a variety of activities, from cooking and food preparation, to fighting, eating, flirting, and sleeping, as in Vincenzo Campi’s *Kitchen* of the 1580s.”

Missing from Campi’s detailed painting, however, is any depiction of a bucket, sink, or soap for handwashing and cleaning the utensils, knives, or tables. The provision and frequent use of such cleaning materials would have reduced the risk for infection from foodborne pathogens. If the kitchen workers or their employers experienced gastrointestinal or skin infections, they would have been unlikely to blame their working conditions. They could not have heard about shiga-toxin producing *Escherichia coli*,* Shigella*, *Salmonella*, *Campylobacter*, and *Cyclospora cayetanensis* as causes of foodborne illnesses or infections. It was not until the second half of the 1600s that scientists such as Robert Hooke and Anton van Leeuwenhoek built microscopes and observed and recorded microorganisms. It then took more than 150 years after those observations that causal links were made between such microorganisms and disease.

Contemporary kitchens with their gleaming counters, appliances for storing and cooking food, and sinks and cleaning products are not likely to inspire artists to depict such a rich, colorful scene as the one Campi captured on his canvas. Nonetheless, while we may think our kitchens are free from all of the unseen hazards in Campi’s *Kitchen*, we still face the same risks for zoonotic infections innocently depicted by Campi. Even with our modern kitchen appliances and comparatively advanced knowledge regarding risk for disease, it is still possible to become ill from eating contaminated or unsafe foods and by inappropriately storing and preparing food.
